# Supplementary figures and images for: Selection and Validation of Reference Genes for RT-qPCR Analysis in Aegilops tauschii (Coss.) under Different Abiotic Stresses
Source: Int J Mol Sci. 2021 Oct 13;22(20):11017. doi: 10.3390/ijms222011017 (PMC8541341; doi:10.3390/ijms222011017)

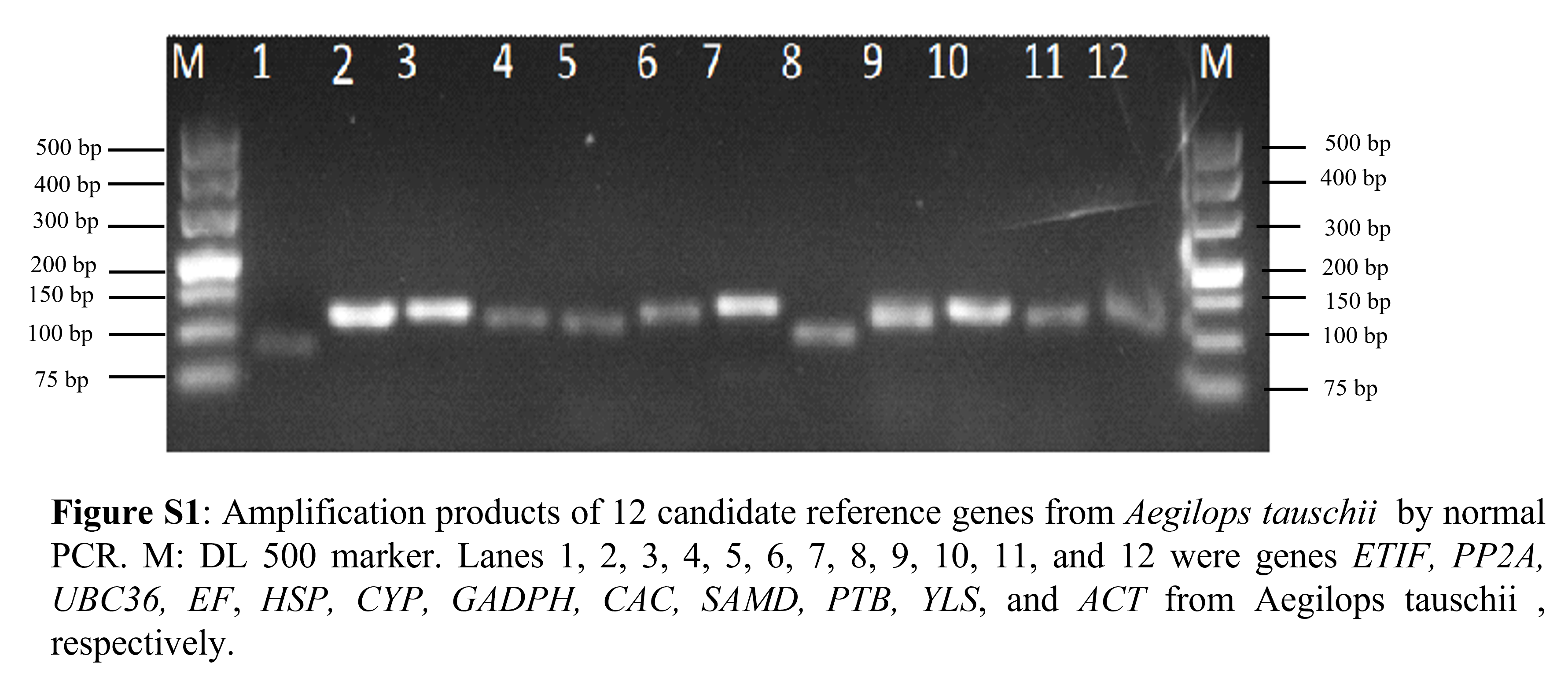

Supplement: Supplementary file 1 [file ijms-22-11017-s001.zip › ijms-1360880-supplementary.png]
